# The relevance of central command for the neural cardiovascular control of exercise

**DOI:** 10.1113/expphysiol.2009.051870

**Published:** 2010-08-09

**Authors:** J W Williamson

**Affiliations:** Department of Health Care Sciences, University of Texas Southwestern Medical Center at Dallas, School of Health ProfessionsDallas, TX, USA

## Abstract

This paper briefly reviews the role of central command in the neural control of the circulation during exercise. While defined as a feedfoward component of the cardiovascular control system, central command is also associated with perception of effort or effort sense. The specific factors influencing perception of effort and their effect on autonomic regulation of cardiovascular function during exercise can vary according to condition. Centrally mediated integration of multiple signals occurring during exercise certainly involves feedback mechanisms, but it is unclear whether or how these signals modify central command via their influence on perception of effort. As our understanding of central neural control systems continues to develop, it will be important to examine more closely how multiple sensory signals are prioritized and processed centrally to modulate cardiovascular responses during exercise. The purpose of this article is briefly to review the concepts underlying central command and its assessment via perception of effort, and to identify potential areas for future studies towards determining the role and relevance of central command for neural control of exercise.

The neural control of the circulation during exercise has been investigated since the late 1800s (Johansson, 1893). Convincing data have emerged demonstrating that cardiovascular responses during exercise are governed by both central (i.e. central command; Gandevia *et al.* 1993) and peripheral mechanisms (i.e. exercise pressor reflex) and their interactions with the arterial baroreflexes (Gallagher *et al.* 2006). Although many studies have sought to explore the specific roles and contributions of these mechanisms, the complexity of the human cardiovascular control system and the redundancy between central and peripheral components have often made interpretation difficult. As identified by Mitchell (1990) over 20 years ago, the relative importance of central command and exercise pressor reflex components in determining responses to exercise is dependent upon the type of exercise (static or dynamic), the intensity of exercise, the time after onset of exercise (immediate, steady state, exhaustion, etc.) and the effectiveness of blood flow in meeting the metabolic needs of the contracting muscles. Accordingly, the primary purpose of this brief review is not to contrast central command with the exercise pressor reflex or compare their interactions with the arterial baroreflexes, but instead to revisit the concept of central command and its assessment as related to findings outside the field of exercise science.

While defining central command is seemingly a perfunctory ritual for discussion purposes, the way in which one defines and uses the term ‘central command’ can have a primary role in determining its perceived relevance. Most would agree that the concept of central command involves descending neural signals from higher brain centres, originally defined as ‘cortical irradiation’, capable of influencing cardiovascular responses during exercise (Krogh & Lindhard, 1913). The majority of investigations involving study of central command have typically defined central command as a ‘feedforward mechanism involving parallel activation of motor and cardiovascular centres’ (Goodwin *et al.* 1972). As such, numerous animal studies investigating central command have employed direct stimulation of brain regions, namely subthalamic regions, capable of generating both motor and cardiovascular responses (Eldridge *et al.* 1985; Waldrop *et al.* 1996). Innovative work by Green *et al.* (2007) and Green & Paterson (2008)) in humans has futher defined midbrain neurocircutry involving the periaqueductal grey. Such studies have provided valuable information regarding both the neural pathways and the influence of descending central signals on the autonomic system and the neural control of the circulation. However, central command has been associated with effort-related cognitive processes involving less well-defined higher cortical regions. Given the lack of direct measures of neural activity from these cortical regions, the magnitude of the central command response has been assessed using an individual's perception of effort or effort sense during exercise, independent of workload or force production (Mitchell, 1990). The rating of perceived exertion (RPE) scale (Borg, 1973) has been widely used to assess the level or magnitude of central command, yet the relationship between central command and rating of perceived exertion has not been clearly defined.

Determination of the specific sensations that drive perception of effort during exercise, as well as their potential influence(s) on central command, has proven more difficult in the interpretation of human research studies. Perception of effort has been associated with somatosenory signals (e.g. from skeletal muscles, heart and lungs; Amann *et al.* 2008), neurocognitive mechanisms (e.g. cognitive ability, environment, experience, knowledge of exertional cues and exercise end-point; Faulkner & Eston, 2008), general discomfort, pain, thermal stress and thirst (Cabanac, 2006), as well as psychobiological factors such as depression and neuroticism (Morgan, 1994). In addition, based on more recent findings from Poulet & Hedwig (2007), evidence was presented that perception of effort can be driven by a corollary discharge from motor to sensory centres during exercise (Marcora, 2009). In concept, the corollary discharges do not generate movement, but may interact with self-generated sensory signals. There remains controversy concerning the specific factors and/or sensations associated with perception of effort that may influence autonomic regulation of cardiovascular function during exercise. From a system perspective, the central integration of afferent sensory feedback is designed to support different types of physical activity that can have different goals, contexts, time courses and exertional levels, each requiring a unique response to meet the body's physiological needs (Craig, 2006).

It is clear that perception of effort represents a very complex interaction involving multiple feedback signals (Fig. 1), yet it has been used as an index of central command. While this does not suggest that ratings of perceived exertion are an inappropriate measure of central command, it does raise questions regarding the potential involvement of feedback mechanism(s) in determining the magnitude of central command. Although central command functions effectively in a feedforward role, it is still possible that central command could also involve conventional feedback mechanisisms (Rowell *et al.* 1996). Multiple feedback signals integrated in perception of effort can affect the cardiovascular system independent of exercise (e.g. pain). It is possible that some of these signals may be able to alter cardiovascular responses in concert with central command (Fig. 1, path A), while others may function independent of central command (Fig. 1, path B). Furthermore, there may be similarities in the central integration of signals capable of modulating both central command during exercise and non-exercise-related ‘cortical modulation of the cardiovascular system’ (Verberne & Owens, 1998). While there may not be definitive answers to these issues, they do merit further discussion in assessing the relevance of central command.

**Figure 1:**
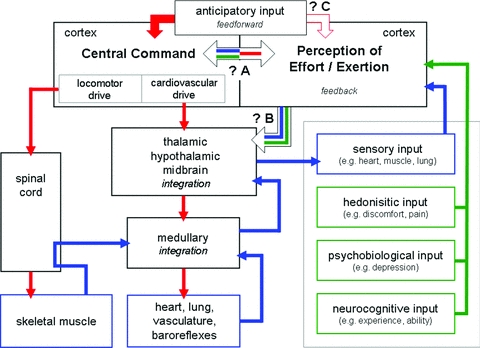
Schematic diagram to show the potential interactions between central command and perception of effort or exertion The red arrows represent the central command pathways (efferent), while the blue (sensory afferent) and green arrows indicate feedback pathways that may influence perception of effort and central command. The open arrows (labelled A, B and C) denote some of the unresolved issues regarding interactions between perception of effort and central command, as follow. Can perception of effort modulate central command or vice versa (A)? Can perception of effort (or specific inputs) influence cardiovascular responses independent of central command during exercise (B)? Can anticipation (as a feedfoward stimulus) modify perception of effort (C)?

## Central command and feedforward control

From an engineering perspective, there are several aspects of a feedforward component in a control system. First, a feedforward component receives its information from a source external to the system and then acts to modify the system before the external influence has a chance to affect the system. In the case of central command, simply the anticipation (e.g. of an increased effort required to ascend a hill) can stimulate a centrally generated command signal which elicits a parallel activation of motor and cardiovascular centres. Second, an effective feedforward component should have the capacity to anticipate the effect of perturbations on the system accurately and send an appropriate signal. For example, as one approaches a hill, centrally mediated increases in heart rate, breathing and muscle activity would become evident prior to the ascent. In most situations, the cardiovascular and motor responses are closely matched to the exercise intensity (i.e. workload, force production), and central command certainly contributes to this response in a feedforward capacity. However, a third point regarding systems control is that even complex feedforward mechanisms cannot always provide perfect compensation. If the anticipated cardiovascular adjustments needed to ascend the hill were underestimated (and not corrected), performance could be compromised. Eventually, feedforward errors will occur and accumulate, resulting in a mismatch between cardiovascular responses and metabolic demand.

To help account for potential feedforward errors, the system also integrates somatosensory signals via feedback mechanisms. As noted previously, the exercise pressor reflex serves as a primary feedback component to correct errors during exercise. Somatosensory signals (e.g. chemical and mechanical) from the working muscle are transmitted to areas of cardiovascular control within the medulla (Mitchell *et al.* 1983) and to regions of the motor cortex (Martin *et al.* 2008). However, these same sensory signals have been implicated in perception of effort (Amann *et al.* 2008). The role of this somatosensory feedback, as well as other factors, in determining perception of effort and/or the level of central command during exercise has been debated (Amann *et al.* 2008; Marcora, 2009). Do the sensations from the working limbs influence perception of effort and modify central command? This question has important implications, in that if somatosensory feedback is involved in modulating the level of central command, then it would suggest that central command (or another effort-related central mechanism working independently from central command) could also function as a feedback component in the overall scheme of cardiovascular regulation during exercise.

## Central command and feedback control

Control systems which incorporate both feedfoward and feedback components can significantly improve system performance over single-component systems (Heylighen & Joslyn, 2001). While a feedforward component acts before an external pertubation has a chance to affect the system, a feedback component typically responds to an internally generated signal(s) and offers error-controlled regulation. Feedback control can be very effective when deviations (e.g. mismatch between blood flow and metabolism) appear gradually or increase slowly, giving the controller the chance to intervene while the deviation is still minimal. Thus, in steady-state conditions, with a very precise feedback component, systems can often work purely in feedback mode. In the case of central command, somatosensory signals arising from the working muscles may provide a feedback signal capable of influencing central command via alterations (or modifications) of perception of effort or effort sense (Amann *et al.* 2008). On the contrary, there are data demonstrating that perceived exertion or effort sense can be modulated to evoke changes in cardiovascular responses independent of changes in muscle afferent input or motor activity (Morgan *et al.* 1973; Thornton *et al.* 2001; Williamson *et al.* 2001, 2002). These studies used hypnosis during exercise to influence a subject's perceived exertion without altering the afferent signals from the working muscles (i.e. cycling at a constant workload). In this scenario, hypnosis was able to modify central neural cardiovascular control mechanisms through alterations in perception of effort.

Perception can be defined as the simultaneous entry of several afferent messages, including those retrieved from memory, into consciousness (Cabanac, 2006). Ratings of perceived exertion have often included sensations related to general discomfort, pain, thermal stress and thirst (Cabanac, 2006). Although exercise scientists have long used ratings of perceived exertion as an index (indirect measure) of central command, should ratings of hedonicity (e.g. pleasant/unpleasant, comfortable/uncomfortable, painful/not painful) be included within the context of central command? If so, this would certainly expand the role and scope of central command beyond that of being exclusively a feedforward component. If not, as these hedonistic factors can modulate cardiovascular responses during exercise (e.g. pain), should an additional ‘central feedback’ component be more explicitly recognized or acknowledged along with central command and the exercise pressor reflex in discussions pertaining to the neural control of the circulation during exercise? Before reaching any immediate conclusions, it may be worthwhile to consider a few findings more closely associated with the behavioural neurosciences or psychobiology which may have relevance to the field of exercise science. Some of the same signals and inputs relevant to the autonomic regulation of the cardiovascular system during exercise are also related to what could be termed cortical modulation of the cardiovascular system (Verberne & Owens, 1998) or, as termed in this review, cortical cardiovascular modulation.

## Central command or cortical cardiovascular modulation

While an operational definition has been provided for central command, cortical modulation of the cardiovascular system (Verberne & Owens, 1998) can be defined as the involvement of the cerebral cortex in cardiovascular control mechanisms (not necessarily associated with exercise). Through interaction with the autonomic nervous system and modulation of sympathetic and parasympathetic activity, both central command and cortical cardiovascular modulation appear to be very similar in their functions, one exception being that central command is associated with perceived exertion or effort during exercise and a parallel activation of motor and cardiovascular systems. With regard to the latter point, it has previously been reported that central command, as assessed by ratings of perceived exertion, can evoke cardiovascular changes during exercise, independent of motor activation (Williamson *et al.* 2006). In other words, a ‘central motor command’ component can be uncoupled from a ‘central cardiovascular command’ component. Furthermore, brain regions involved in central cardiovascular command (Williamson *et al.* 2006; Green & Paterson, 2008) and cortical cardiovascular modulation (Verberne & Owens, 1998) include both the insular cortex and the medial prefrontal cortex; these regions have been implicated in cortically evoked circulatory responses. Given the striking similarity in cortical neuroanatomy, is the central integration and processing of information involved in cardiovascular modulation during exercise (e.g. central command) similar for non-exercise-related cortical cardiovascular modulation (e.g. muscle pain) or do they engage distinct neural networks?

Central command and muscle pain do share common neural substrate, it that both activate regions of the insular cortex and anterior cingulate cortex (Williamson *et al.* 2002; Macefield *et al.* 2007). However, it has been proposed that central command is involved in the central modulation of exercise-induced muscle pain (Ray & Carter, 2007). It was found that central command modulates the perception of muscle pain during exercise, in that pain perception was increased in the absence of central command. In this instance, is central command serving as a ‘distracting stimulus’ to modify the pain response? Another possibility is that central command may be suppressing muscle afferent input at the spinal level via release of GABA (Degtyarenko & Kaufman, 2003). To further complicate matters, central command as measured by ratings of perceived exertion can itself be modified by distractive stimuli (e.g. music; Boutcher & Trenske, 1990). In this scenario, attention to the external environment is thought to reduce the awareness of physiological sensations and negative emotions, which can serve to decrease perception of effort. On the contrary, the removal of distracting external information (via sensory deprivation) can increase perception of effort. It has been suggested that preconscious processing can selectively filter the information available for conscious awareness, leading to effort sense (or perception of effort), from which conscious decisions are made regarding the continuation of exercise (Hampson *et al.* 2001). The implication is that central command has the capacity to modify or modulate sensory information. It would appear that another measure of central command (e.g. skin sympathetic nerve activity; Vissing & Hjortso, 1994; Ray & Wilson, 2004) could be of value in assessing and clarifying the complex interactions between central command and the specific neural signals and sensations associated with ratings of perceived exertion.

## Summary

Highly regulated neural inputs are critical to maintaining normal cardiovascular function. Operationally, central command is typically associated with perception of effort during exercise, while cortical cardiovascular modulation is more commonly associated with non-exercise conditions, such as emotion, stress and pain. The neuroanatomical infrasturcure used by central command for cardiovascular control during exercise is basically the same as that employed for central modifications of the cardiovascular system in non-exercise conditions. While the mental processes and behaviours involving sensation, perception, motivated behaviours and control of movement can be included under the umbrella of the behavioural neurosciences, they can also be studied during exercise (e.g. exercise neuroscience?). With regard to the ‘role of the cortex in the neural control of the circulation’, is this a situation where the disciplines of exercise neuroscience and behavioural neuroscience have been looking at different sides of the same coin? Some may argue the differences, yet there are certainly numerous similarities between central command and cortical cardiovascular modulation that should not be overlooked. Whether some of the more clearly defined mechanisms underlying behavioural sciences can be applied to central command during exercise remains unknown.

The association between central command and perception of effort has raised questions. An individual's perceived exertion (or effort) during muscular exertion appears to integrate all afferent sensory inputs along with hedonistic sensations, which can also involve motivation (Cabanac, 2006). As such, central command appears to have the capacity to function as a feedback component. The magnitude of central command within the central nervous system is likely to be the result of complex interaction of feedforward as well as various feedback mechanisms (Amann *et al.* 2008). Identification of the specific roles of the various feedback mechanisms remains problematic in human investigation. As one feedback component is removed, the system may compensate by altering the influence of other inputs. Furthermore, modification of neural control systems may also occur in various disease states; the role of central command can be altered by myocardial infarction (Koba *et al.* 2006). Central command is a critical part of the cardiovascular control system, but should it be viewed as more than a feedforward component? Only through a clearer understanding of the role of central command in the integration of sensory information can we define more completely the relevance of central command for the neural control of exercise.
